# Milk lymphocyte profile and macrophage functions: new insights into the immunity of the mammary gland in quarters infected with *Corynebacterium bovis*

**DOI:** 10.1186/s12917-021-02989-5

**Published:** 2021-08-25

**Authors:** Vitória M. Silva, Marina T. Souza, Maiara G. Blagitz, Fernando N. Souza, Camila F. Batista, Alexandre J. Alves, Artur C. C. Fernandes, Eduardo M. R. Sanchez, Carla M. Ordinola-Ramirez, Luciana da Costa, Alice M. M. P. Della Libera

**Affiliations:** 1 Núcleo Aplicado à Produção e Sanidade da Glândula Mamária, Departamento de Ciências Veterinárias, Centro de Ciências Agrárias, Universidade Federal da Paraíba, 58397-000 Areia, Brazil; 2 Veterinary Clinical Immunology Research Group, Departamento de Clínica Médica, Faculdade de Medicina Veterinária e Zootecnia, Universidade de São Paulo, Av. Prof. Dr. Orlando Marques de Paiva, 87, 05508-270 São Paulo, Brazil; 3 Programa de Pós-Graduação em Ciência Animal, Universidade Federal da Paraíba, 58397- 000 Areia, Brazil; 4 Departamento de Salud Publica, Facultad de Ciencias de La Salud, Universidad Nacional Toribio Rodriguez de Mendoza de Amazonas, 01000 Chachapoyas, Peru; 5 Laboratório de Sorologia e Imunobiologia, Instituto de Medicina Tropical, Universidade de São Paulo, 05403-000 São Paulo, Brazil; 6 Department of Preventive Veterinary Medicine, College of Veterinary Medicine, The Ohio State University, Columbus, 43210 USA

**Keywords:** Immune response, Phagocytes, T lymphocyte, Mastitis, Milk, Dairy cow

## Abstract

**Backgrounds:**

The present study explored the viability of bovine milk macrophages, their intracellular production of reactive oxygen and nitrogen species (RONS), and their phagocytosis of *Staphylococcus aureus*, as well as the profile of lymphocytes, from healthy udder quarters and udder quarters infected by *Corynebacterium bovis*. The study included 28 healthy udder quarters from 12 dairy cows and 20 udder quarters infected by *C. bovis* from 10 dairy cows. The percentages of macrophages and lymphocytes were identified by flow cytometry using monoclonal antibodies. Macrophage viability, RONS production, and *S. aureus* phagocytosis were evaluated by flow cytometry.

**Results:**

Milk samples from quarters infected with *C. bovis* showed a lower percentage of macrophages but an increased number of milk macrophages per mL and a higher percentage of macrophages that produced intracellular RONS and phagocytosed *S. aureus*. No effect of *C. bovis* infection on macrophage viability was found. Udder quarters infected by *C. bovis* showed a higher percentage of T cells and CD4^+^ T lymphocytes, but no effect was found on the percentage of CD8^+^ CD4^−^ T, CD8^−^ CD4^−^ T, or B lymphocytes.

**Conclusions:**

Thus, our results corroborate, at least in part, the finding that intramammary infections by *C. bovis* may offer protection against intramammary infections by major pathogens.

**Supplementary Information:**

The online version contains supplementary material available at 10.1186/s12917-021-02989-5.

## Background

*Corynebacterium bovis* is one of the bacteria most commonly isolated from aseptically collected bovine milk samples worldwide that are subjected to microbiological examination to identify the pathogens that cause bovine mastitis [1–4]. Despite its high prevalence in the etiology of intramammary infections in cattle, *C. bovis* is considered a minor mastitis pathogen with limited clinical significance [5]. From another point of view, it has been considered part of the udder core microbiota with potential protective role against dysbiosis [6–8]. This bacterium colonizes the teat apices [9, 10], teat canal [11] but can also be isolated from the teat cistern, gland cistern, and mammary parenchyma [12]. Although the milk somatic cell count (SCC), which is an inflammatory indicator widely used in the diagnosis of bovine mastitis, is relatively low in milk samples from udder quarters from which *C. bovis* is isolated, their SCC value is still usually higher compared to healthy udder quarters [1, 13, 14]. No effect on milk production [4, 14] or on the percentage of fat, protein, casein, and total solids was described [4].

*C. bovis* is of interest to mastitis researchers because quarters infected with this bacterium are less likely to become infected with other, more pathogenic bacteria [6, 13, 15–19]. Previous studies conducted by our research group investigated the functions of milk neutrophils (e.g., viability, phagocytosis and intracellular reactive oxygen species production) in udder quarters infected with *C. bovis* [20, 21]. Thus, to deepen our understanding of the mammary gland immunity in *C. bovis*-infected udder quarters, this study aimed to investigate the lymphocyte profile of milk and the function of milk macrophages of healthy mammary glands compared to udder quarters naturally infected with *C. bovis*.

## Results

**Fig. 1:**
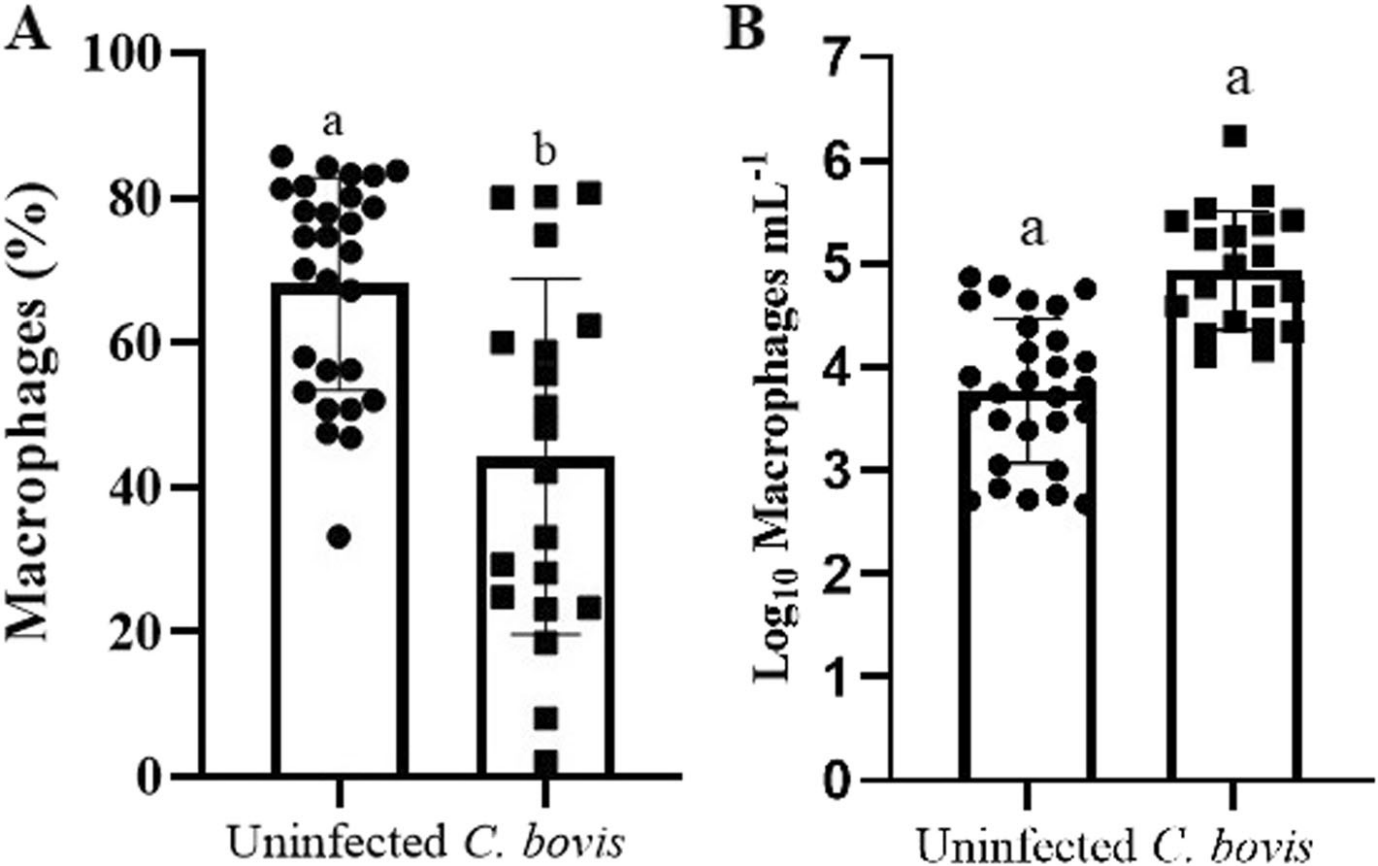
The antidromic trend of the percentage and number of milk macrophages in *Corynebacterium bovis-*infected quarters. Percentage (mean ± standard error) of milk macrophages (**A**) and the number of milk macrophages per mL (**B**) in healthy mammary quarters vs. mammary quarters infected with *C. bovis*. Different letters indicated *P* ≤ 0.05

Here, no statistical difference on days in milk between uninfected (192.8 ± 19.8) and *C. bovis*-infected (223.6 ± 17.5) quarters was found (*P* = 0.53). The logarithmic milk SCC in *C. bovis*-infected quarters was higher than healthy ones (*P* < 0.0001). The percentage of milk macrophages was higher in healthy udder quarters (68.11 ± 2.78; *P* = 0.0008) than in quarters infected by *C. bovis* (44.25 ± 5.49; Fig. 1A), nonetheless the logarithmic number of macrophages per mL was higher in *C. bovis*-infected quarters (4.93 ± 0.13) than in healthy ones (3.77 ± 0.13, *P* < 0.0001; Fig. 1B). The percentages of viable milk macrophages (Annexin V^−^/PI^−^; Healthy quarters: 41.73 ± 3.30, *C. bovis*-infected quarters: 40.29 ± 4.33, *P* = 0.79) and apoptotic milk macrophages (Annexin V^+^/PI^−^; Healthy quarters: 39.20 ± 3.58, *C. bovis-*infected quarters = 31.86 ± 4.59, *P* = 0.17) did not differ by infection status.

**Fig. 2:**
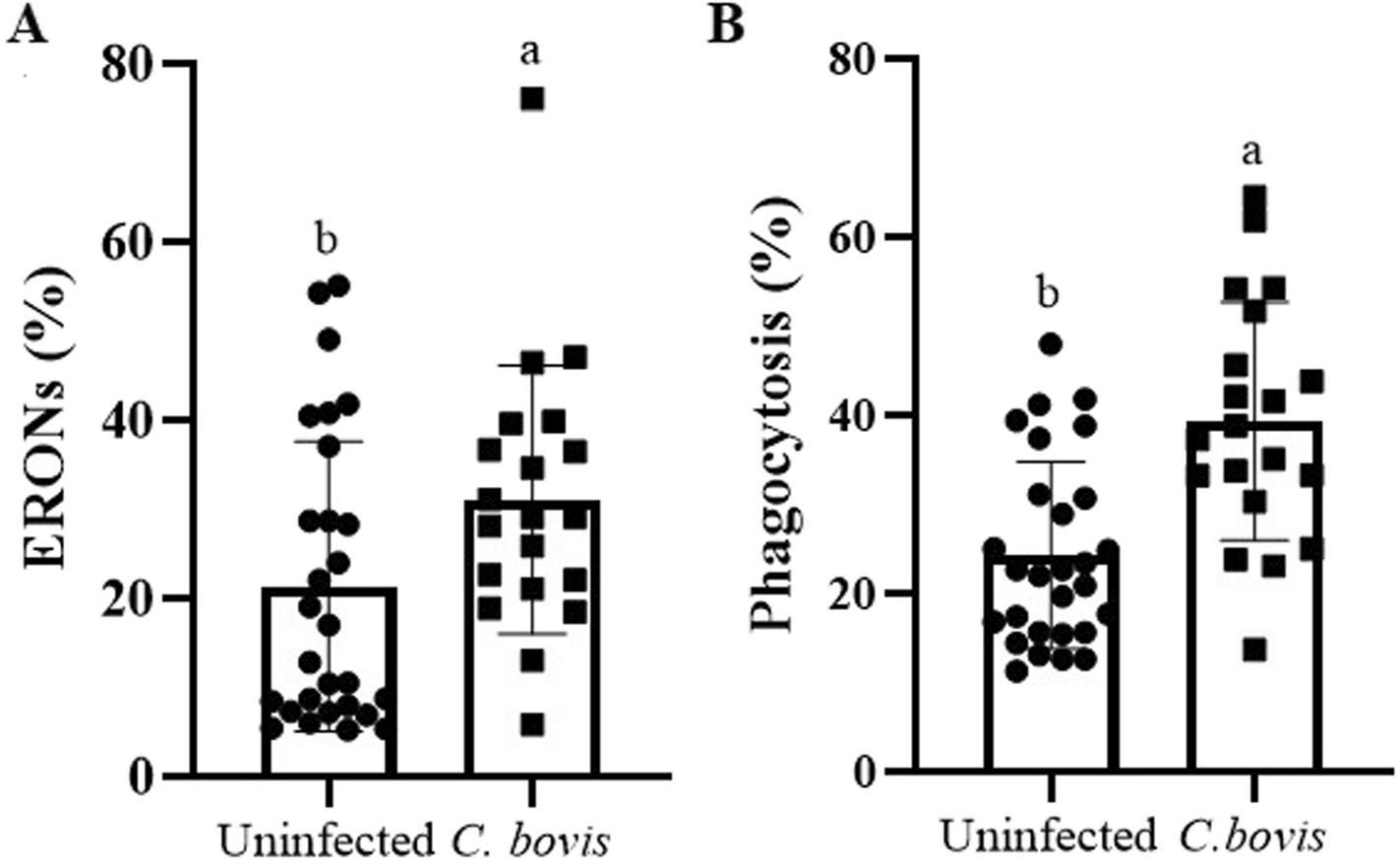
*Corynebacterium bovis-*infected quarters was associated with a higher phagocytosis and intracellular RONS by milk macrophages. Percentage (mean ± standard error) of milk macrophages that produced RONS (**A**) and phagocytized *S. aureus* (**B**) in healthy mammary quarters vs. mammary quarters infected with *C. bovis*. RONS: reactive oxygen and nitrogen species. Different letters indicated *P* ≤ 0.05

The percentage of milk macrophages that produced RONS was higher in udder quarters infected by *C. bovis* (31.12 ± 3.37; *P* = 0.035) than in those considered healthy (21.34 ± 3.082; Fig. 2A). However, the intensity of RONS production by milk macrophages did not differ between the udder quarters infected with *C. bovis* (1168 ± 177.3; *P* = 0.28) and healthy udder quarters (978.5 ± 72.39; Supplemental Material 2). Similarly, the percentage of macrophages that phagocytosed *S. aureus* was higher in udder quarters infected with *C. bovis* (39.36 ± 2.99; Fig. 2B) than in those considered healthy (24.32 ± 1.98; *P* = 0.0001). However, the intensity of phagocytosis did not differ between the healthy udder quarters (84.30 ± 6.89) and those infected with *C. bovis* (71.79 ± 8.00; *P* = 0.22; Supplemental Material 2).

**Fig. 3:**
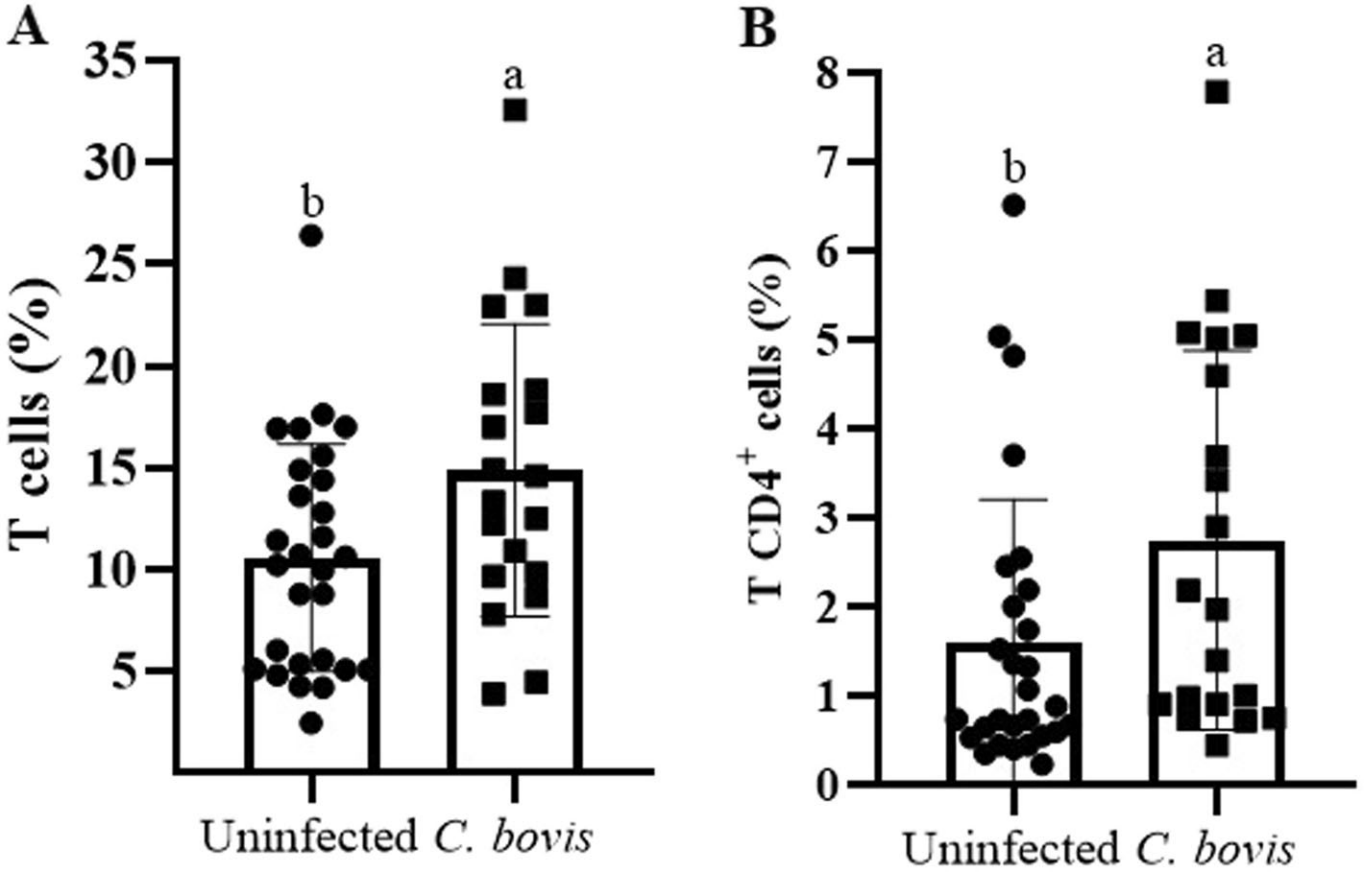
*Corynebacterium bovis-*infected quarters had a higher percentage of T cells, especially T CD4^+^ lymphocytes. Percentages (mean ± standard error) of T lymphocytes (**A**) and CD4^+^ T lymphocytes (**B**) in healthy mammary quarters and mammary quarters infected with *C. bovis*. Different letters indicated *P* ≤ 0.05

*Corynebacterium bovis*-infected udder quarters had higher percentages of milk T cells (CD3^+^; *P* = 0.02, Fig. 3A) and T CD4^+^ lymphocytes (*P* = 0.02, Fig. 3B) than uninfected ones, but no effect on the percentage of CD8^+^ CD4^-^ T cells, CD8^-^ CD4^-^ T lymphocytes, or B cells (CD21^+^) was found (Supplemental Material 1).

## Discussion

Here, we used milk samples from healthy and *C. bovis*-infected udder quarters from different dairy cows as well as from the same cow. In this regard, we should highlight that the immune response against *C. bovis* is primarily restricted to the *C. bovis*-infected quarter [5, 6, 20], and the interdependence of udder quarters was also regarded in the statistical analysis of the present study, as previously suggested [22, 23].

Although it is difficult to draw consistent conclusions about the role of *C. bovis* on bovine udder health, there is an increasing evidence that *C. bovis* is part of udder microbiota [7, 11], which could modulate the local immune response [7, 24]. Thus, a recent study published by Porcellato et al. [7] using milk microbiome analysis associate the presence of the genera *Corynebacterium* in milk samples with a protective role against bovine mammary gland dysbiosis. In another study that investigated the association of teat canal microbiome analysis and mastitis susceptibility and udder inflammation, *Corynebacterium* identification was negatively correlated with milk SCC [11]. Additionally, the *Corynebacterium* genera dominate the bacterial populations of teat apices from healthy quarters [9]. Altogether, although the mechanisms behind these phenomena remain to be fully determined, our findings indicated that *C. bovis* could positively impact udder health by optimizing mammary gland immunity. In this scenario, for the first time, we observed an augment in the percentage of T cells, especially T CD4^+^ cells, as well as a higher percentage of milk macrophages that phagocytosed *S. aureus* and produced RONS in *C. bovis-*infected quarters.

The ability of cows to resist the establishment of new intramammary infections or to overcome existing intramammary infections depends on the efficiency the mammary gland immunity. In this context, macrophages play a critical role in the initiation of the innate immune response of the host in case of bacterial (i.e., *Streptotoccus uberis*) invasion into the mammary gland [25, 26], beyond their role on adaptative immunity, such as antigen processing and presentation [27]. Similarly, T CD4^+^ lymphocytes have been associated with clearance activity against several bacterial pathogens [28–30]. Furthermore, cows having higher frequency of T CD4^+^ lymphocytes than T CD8^+^ lymphocytes in their mammary gland secretions appear to be more resistant to mastitis [31]. Overall, these findings support, at least in part, the potential protective role of *C. bovis* in the bovine udder.

In the present study, no perturbation on CD8^+^ T lymphocytes was found in quarters in which *C. bovis* was isolated, although the percentage of this lymphocyte subpopulation increased during staphylococcal and streptococcal intramammary infections [32]and may play a suppressive and cytotoxic role in the mammary gland [27, 33, 34]. In addition, the percentage of CD4^−^ CD8^−^ T lymphocytes, mainly represented by γδ T cells [35] was not affected by *C. bovis*. Furthermore, the macrophage apoptosis did not appear to be involved in the inflammatory response during *C. bovis*-infection.

Thus, our results corroborate other studies that found a high correlation between isolation of *C. bovis* from aseptically collected milk samples and the reduction in the occurrence of intramammary infection by other pathogenic bacteria [6, 13, 16–18], although there is no consensus on this assertion [36–39]. In addition to our data regarding the lymphocytic and functional profile of milk macrophages, other factors may be related to the potential protective effect of intramammary infection by *C. bovis*, such as inhibition of growth by competition, bacterial antagonism, increased activity of milk neutrophils, and the presence of plasmocytes in the parenchymal tissue and the teat apices of quarters colonized with *C. bovis* [6, 16, 21, 40].

Furthermore, macrophages represent the first line of defense of the mammary gland against invasive pathogens because they make up the predominant leukocyte population in healthy udder quarters [27, 30, 41]. Although the proportion of macrophages usually decreases during intramammary infections due to the rapid and massive influx of neutrophils into the inflammatory site [19, 42], macrophages still represented the predominant leukocyte population in quarters infected by *C. bovis* in the present study. Besides that, an increase in the number of macrophages per mL was observed in *C. bovis*-infected quarters.

## Conclusions

The present study by evaluating the phagocytosis of milk macrophages and the percentage of T lymphocytes corroborates with previous findings indicating that isolation of *C. bovis* from aseptically collected milk samples could be associated with a protection against the major mastitis pathogens, such as *S. aureus*. However, the mechanisms behind these findings need to be further in-depth investigated, which also consider the role of *C. bovis* in the complexity of mammary gland microbiota and how exactly this microorganism acts to promote udder health.

## Methods

### Animals and sampling

The present study used 48 udder quarters from 18 clinically healthy Holstein dairy cows (daily milk yield = 24.02 ± 1.97) on their second and third lactation, collected at different lactation stages from a commercial herd. Immediately postpartum dairy cows were not included. From these dairy cows, we selected 20 *C. bovis*-infected quarters from 10 dairy cows and 28 culture-negative control quarters from 12 dairy cows with no abnormal secretions in the strip cup test and a quarter SCC lower than 1 × 10^5^ cells/mL, as the threshold for SCC described by Bansal et al. [43] for uninfected quarters.

First, the strip cup test was carried out to detect potential clinical mastitis cases. Then, a single milk quarter sample (about 4 mL) was aseptically collected for bacteriological analysis as recommended by National Mastitis Council [44]. Furthermore, quarter milk samples for SCC measurements (40 mL) were taken in sterile tubes containing micropellets of Bronopol (2-bromo-2-nitroprane-1,3-diol). Finally, 1 L of milk samples for the evaluation of monocyte/macrophage function and lymphocyte profile were collected. Until milk samples arrived at the laboratory, they were maintained at 4 °C. Subsequently, quarter milk samples for bacteriological examination were retained at -20 °C until the analysis.

Afterwards, all samples were randomized and codified, and milk analyses were carried out without knowledge of the status of the udder quarter. All methods were carried out in accordance with relevant guidelines and regulations.

### Bacteriological culture

The bacterial analysis was carried out by culturing 0.01 mL of each milk quarter sample on 5 % ovine blood agar plates. The plates were incubated for 72 h at 37 °C, followed by Gram staining, observation of colony morphology, and biochemical testing [45]. A milk sample was considered culture positive when the growth of ≥ 4 pure *C. bovis* colonies was detected [20]. A sample was considered culture negative if there was no growth (no colony from a 0.01 mL sample; < 100 colony-forming units per mL).

### Determination of the somatic cell count

The SCC was determined with the automated somatic cell counter Somacount 300 (Bentley Instruments, Chaska, MN, USA).

### Separation of milk cells

Milk cells were separated as previously described by Blagitz et al. [20]. Briefly, 1 L of milk was diluted in 1 L of phosphate-buffered saline solution (PBS). Centrifugation at 1000×g was performed for 15 min, and the fat layer and the supernatant were discarded. The cell pellet was then washed again with 30 mL of PBS solution and centrifuged at 400 × g for 10 min. This cell pellet was resuspended in 1 mL of RPMI-1640 cell culture medium (R7638, Sigma Aldrich, USA) supplemented with 10 % fetal bovine serum (Cultilab, Brazil), and then the cells were counted in a Neubauer chamber. Cell viability was initially assessed by exclusion using Trypan blue. The cells present in the milk were then resuspended in cell culture medium containing 10 % fetal bovine serum at a concentration of 2 × 10^6^ mL^− 1^ viable cells.

### Enumeration of lymphocyte subpopulations

The enumeration of lymphocyte subsets was performed as previously described [30] with some slight modifications. Briefly, the cells were washed with PBS and stained with a combination of CD3, CD4, and CD8 (tube 1) and for CD21 (tube 2) for 30 min at room temperature. The lymphocyte subpopulations were identified based upon their cytoplasmatic granularity and mean fluorescence intensity following two-step fluorescence immunolabeling with primary anti-bovine monoclonal antibodies (mAbs) and the secondary antibody (Ab) coupled to the long-wavelength fluorescent probes (Supplemental Material 3). After washing with PBS, the cells were incubated for 30 min at room temperature with the secondary Abs. The cells were subsequently washed with PBS and quickly evaluated by flow cytometry (FACSCalibur, BD Bioscience, San Jose, CA, USA). Here, 20,000 milk cells, excluding most of the cell debris, were examined in each quarter milk sample. A single-stained, fluorochrome-conjugated secondary Ab control and unstained control milk samples were also prepared as compensation controls. FlowJo software (TreeStar Inc., Ashland, OR, USA) was used to analyze the data.

### Identification of milk macrophages

The identification of macrophages was carried out as previously described [46]. Initially, the cells were incubated with 1 µL of mouse IgG1 mAb against bovine CD14 (cat. n. MM61A, VMRD, Pullman, WA, USA) for 30 min at room temperature. Immediately after, 1 mL of PBS was added to the specific cytometry tube, and the samples were centrifuged for 8 min at 400 × g. Next, 1 µL of allophycocyanin-conjugated goat anti-mouse IgG1 secondary antibody (cat. n. A10541, Invitrogen, Carlsbad, CA, USA) was added to the samples, which were incubated for 30 min at room temperature. Then, PBS solution (1 mL) was added to the cell suspension, which was centrifuged for 8 min at 400 × g. Lastly, PBS (300 µL) was added to the samples, which were examined by flow cytometry (FACSCalibur, BD Bioscience, San Jose, CA, USA). A single-stained, fluorochrome-conjugated secondary Ab control and unstained control milk samples were also prepared as compensation controls. FlowJo software (TreeStar Inc., Ashland, OR, USA) was used to analyze the data.

### Preparation of *Staphylococcus aureus* stained with propidium iodide

The staining of *Staphylococcus aureus* (ATCC 25,923) with propidium iodide (PI) was done as proposed [47, 48].

### Intracellular production of reactive oxygen and nitrogen species

The intracellular production of RONS was measured by flow cytometry as described [30, 41, 48]. Briefly, 2 × 10^5^ viable milk cells were incubated with 200 µL of 2′,7′-dichlorofluorescein diacetate (DCFH_2_-DA, 0.3 mM, cat. n. D6883, Sigma Aldrich, St. Louis, USA) for 30 min at 37°C. Various types of RONS (hydrogen peroxide, peroxynitrite, nitric oxide, hydroxyl radicals, and peroxyl) oxidize DCFH_2_-DA into 2’,7’-dichlorofluorescein (DCF), which is fluorescent and can be detected in a flow cytometer equipped with a set of standard filters for fluorescein green [49]. After the incubation in DCFH_2_-DA, 2 mL of 3 mM EDTA was added. Macrophages were identified using the CD14 mAb as described above. Finally, the samples were centrifuged at 400×g for 10 min, and the leukocytes were resuspended in 300 µL of PBS and analyzed by flow cytometry.

In the present study, 20,000 cells per sample were examined – most cellular debris was excluded. The readings of the samples were performed in a flow cytometer with argon (excitation 488 nm) and diode lasers (excitation 635 nm) (FACSCalibur, BD Bioscience, San Jose, CA, USA). FlowJo software (TreeStar Inc., Ashland, OR, USA) was used to examine the data. The data are presented as the percentage of macrophages (CD14^+^ cells) that produced RONS (percentage of fluorescent cells), and the geometric mean fluorescence intensity (GMFI) indicated the intensity of RONS production of each cell. The results were corrected for autofluorescence content using nonstained milk cells from milk samples from the same udder quarter.

### Phagocytosis

The phagocytosis assay was performed by flow cytometry using *S. aureus* conjugated with propidium iodide (PI) as previously described [41, 46, 48]. Briefly, 2 × 10^5^ viable milk cells were incubated with 100 µL of PI-conjugated *S. aureus* for 30 min at 37 °C and 900 µL of PBS. Then 2 mL of 3 mM EDTA was added to drastically reduce the number of bacteria adhering to the cell membrane that could be mistakenly considered phagocytized [47, 50]. The macrophages were identified using the CD14 mAb as described above. Finally, the samples were centrifuged at 400×g for 10 min, and the leukocytes were resuspended in 300 µL of PBS and analyzed by flow cytometry.

As above, 20,000 cells per sample were examined, and most cellular debris was excluded. FlowJo software (TreeStar Inc., Ashland, OR, USA) was used to examine the data. The data are presented as the percentage of macrophages (CD14^+^ cells) that phagocytized PI-stained bacteria (percentage of fluorescent cells), and the GMFI indicates the number of bacteria phagocytized by macrophages that phagocytosed *S. aureus* by measuring the fluorescence intensity, which was correlated with the number of phagocytized bacteria per cell. The results were corrected for autofluorescence content using nonstained milk cells from milk samples from the same udder quarter.

### Detection of apoptosis by flow cytometry

Apoptosis of milk macrophages was determined by double staining with annexin-V conjugated to fluorescein isothiocyanate (FITC) and propidium iodide (PI) by flow cytometry analysis using a commercial kit (cat. n. K2350, APOPTEST-FITC, DakoCytomation, Netherlands), as previously described [30, 41, 48]. Initially, 2 × 10^5^ milk cells were resuspended in 100 µL of binding buffer (10 mM HEPES, 150 mM NaCl, 1 mM MgCl_2_ and 1.8 mM CaCl_2_) containing annexin-V FITC and incubated at room temperature for 20 min in the dark. The macrophages were identified using the CD14 mAb as described above. Immediately before the flow cytometry analysis, 5 µL of a PI solution (250 µg mL^− 1^) was added. Cells negative for FITC-stained annexin-V and for PI were considered alive. Cells that were reactive to FITC-stained annexin-V but negative to PI were classified as apoptotic. Again 20,000 cells were examined per sample, and most cellular debris were excluded. FlowJo software (TreeStar Inc., Ashland, OR, USA) was used to examine the data.

### Statistical analysis

The distributions of all variables were analyzed using normal probability plots obtained from the Shapiro-Wilk test. As all data presented high coefficient of variation, we carried out a logarithmic transformation (Log_10_). First, interclass correlation at the cow and quarter levels was calculated to determine the strength of clustering, as previously described by McGraw and Wong [51]. The data were analyzed ANOVA following by the pos-hoc Student-Newnan-Keuls test was applied. The model of mammary quarters and cows nested within cows was considered [23]. Statistical analyses were performed using the statistical software InfoStat (Cordoba, Argentina). The results are presented as the mean ± standard error. The level of significance was set at *P* ≤ 0.05.

## Supplementary Information

**Additional file 1: Supplemental Table 1.** Percentage (mean ± standard error) of B and T lymphocytes in milk samples from healthy mammary quarters infected with *Corynebacterium bovis.*

**Additional file 2: Supplemental Figure 1.**

**Additional file 3: Supplemental Table 2.** Monoclonal antibodies used for immunophenotyping bovine milk lymphocytes by flow cytometry.

## Data Availability

The datasets used and/or analysed during the current study are available from the corresponding author on reasonable request.

## Abbreviations

SCC: Somatic cell count
PBS: Phosphate-buffered saline solution
mAb: Monoclonal antibody
ab: Antibody
PI: Propidium iodide
RONS: Reactive oxygen and nitrogen species
DCFH_2_-DA: 2′,7′-dichlorofluorescein diacetate
DCF: 2’,7’-dichlorofluorescein
GMFI: Geometric mean fluorescence intensity
FITC: Fluorescein isothiocyanate
