# Supplementary material for: Milk lymphocyte profile and macrophage functions: new insights into the immunity of the mammary gland in quarters infected with Corynebacterium bovis
Source: BMC Vet Res. 2021 Aug 25;17:282. doi: 10.1186/s12917-021-02989-5 (PMC8390291; doi:10.1186/s12917-021-02989-5)
Supplement: Supplementary file 1 — Additional file 1: Supplemental Table 1. Percentage (mean ± standard error) of B and T lymphocytes in milk samples from healthy mammary quarters infected with Corynebacterium bovis. [file 12917_2021_2989_MOESM1_ESM.docx]

**Supplemental Material 1**

**Supplemental Table 1.** Percentage (mean ± standard error) of B and T lymphocytes in milk samples from healthy mammary quarters infected with *Corynebacterium bovis*

| **Lymphocyte population** | **Healthy (%)** | ***Corynebacterium bovis*** (%) |
| --- | --- | --- |
| **B lymphocytes (CD21^+^)** | 14.16 ± 1.45^a^ | 14.89 ± 1.30^a^ |
| **T lymphocytes (CD3^+^)** | 10.56 ± 1.06^b^ | 14.87 ± 1.61^a^ |
| **CD4^+^ CD8^-^ T lymphocytes** | 1.60 ± 0.30^b^ | 2.75 ± 0.48^a^ |
| **CD4^-^ CD8^+^ T lymphocytes** | 4.64 ± 0.57^a^ | 6.31 ± 0.76^a^ |
| **CD4^-^ CD8^-^ T lymphocytes** | 4.03 ± 0.67^a^ | 5.51 ± 0.99^a^ |

Different letters indicate *P* < 0.05.
