# Supplementary figures and images for: Milk lymphocyte profile and macrophage functions: new insights into the immunity of the mammary gland in quarters infected with Corynebacterium bovis
Source: BMC Vet Res. 2021 Aug 25;17:282. doi: 10.1186/s12917-021-02989-5 (PMC8390291; doi:10.1186/s12917-021-02989-5)

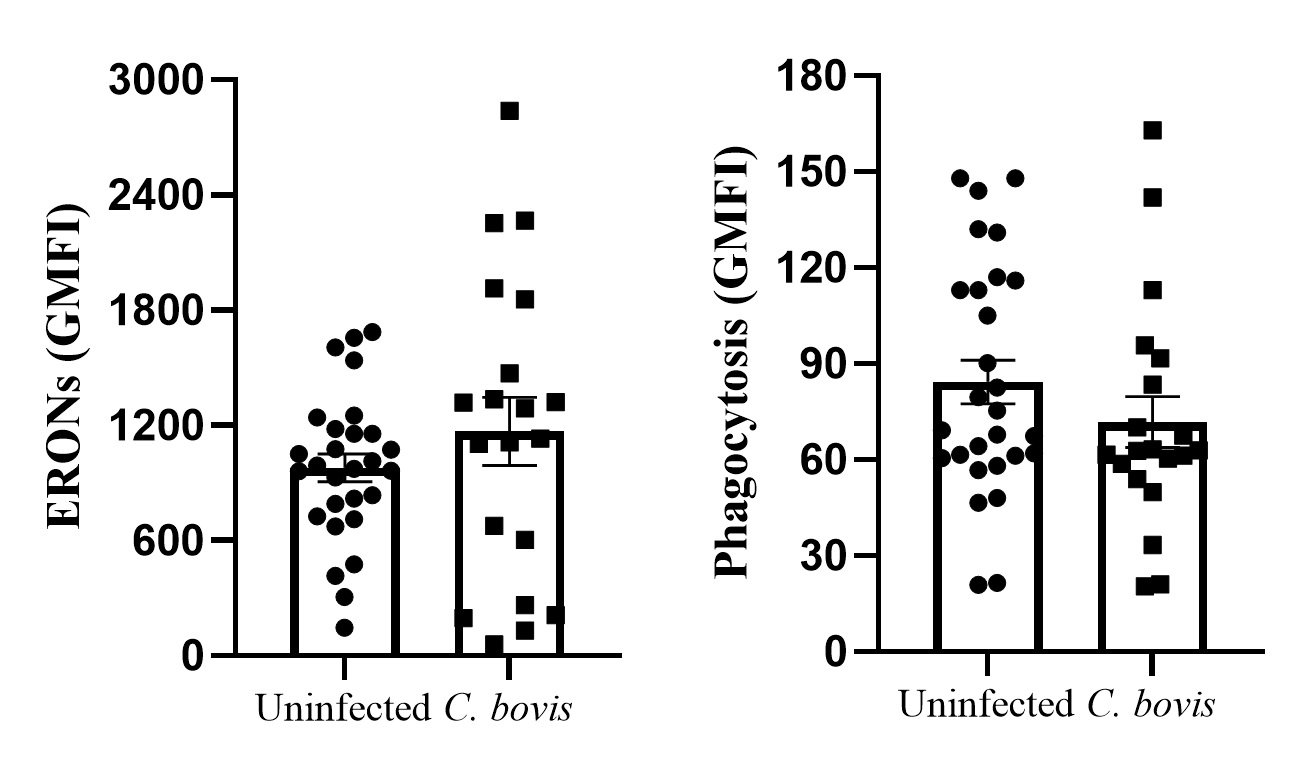

Supplement: Supplementary file 2 — Additional file 2: Supplemental Figure 1. [file 12917_2021_2989_MOESM2_ESM.tiff]
