# Supplementary material for: Milk lymphocyte profile and macrophage functions: new insights into the immunity of the mammary gland in quarters infected with Corynebacterium bovis
Source: BMC Vet Res. 2021 Aug 25;17:282. doi: 10.1186/s12917-021-02989-5 (PMC8390291; doi:10.1186/s12917-021-02989-5)
Supplement: Supplementary file 3 — Additional file 3: Supplemental Table 2. Monoclonal antibodies used for immunophenotyping bovine milk lymphocytes by flow cytometry. [file 12917_2021_2989_MOESM3_ESM.docx]

**Supplemental Material 2**

| Description | Primary antibody | | | | | | | Secondary antibody | | | | | | |
| --- | --- | --- | --- | --- | --- | --- | --- | --- | --- | --- | --- | --- | --- | --- |
|  | Name | Type | Amount | Specificity | Host | Company | Isotype | Name | Type | Amount | Specificity | Host | Company | Isotype |
| T Lymphocyte | MM1A | CD3 | 1 µL | Bovine | Mouse | VMRD^1^ | IgG1 | M32018 | IgG1 – PE-Cy5 | 1 µL | Mouse | Goat | Invitrogen^2^ | IgG1 |
| CD4 T Lymphocyte | ILA11 | CD4 | 1 µL | Bovine | Mouse | VMRD^1^ | IgG2a | M32204 | IGG2a – PE | 1 µL | Mouse | Goat | Invitrogen^2^ | IgG2a |
| CD8 T Lymphocyte | BAQ111A | CD8 | 1 µL | Bovine | Mouse | VMRD^1^ | IgM | M31501 | IgM – FITC | 1 µL | Mouse | Goat | Invitrogen^2^ | IgM |
| B Lymphocyte | BAQ15A | CD21 | 1 µL | Bovine | Mouse | VMRD^1^ | IgM | M31505 | IgM – APC | 2 µL | Mouse | Goat | Invitrogen^2^ | IgM |

**Supplemental Table 2.** Monoclonal antibodies used for immunophenotyping bovine milk lymphocytes by flow cytometry

APC: Allophycocyanin; FITC: fluorescein isothiocyanate; PE: R-Phycoerythrin; PE-Cy5: Phycoerythrin cyanine 5; ^1^VMRD Pullman Inc. Corp^®^, Pullman, WA, USA; ^2^Invitrogen, Carlsbad, CA, USA.
